# Initial non-invasive in vivo sensing of the lung using time domain diffuse optics

**DOI:** 10.1038/s41598-024-56862-0

**Published:** 2024-03-15

**Authors:** Antonio Pifferi, Massimo Miniati, Andrea Farina, Sanathana Konugolu Venkata Sekar, Pranav Lanka, Alberto Dalla Mora, Giulia Maffeis, Paola Taroni

**Affiliations:** 1 Dipartimento di Fisica, Politecnico di Milano, 20133 Milan, Italy; 2 IFN-CNR, Consiglio Nazionale delle Ricerche, Istituto di Fotonica e Nanotecnologie, 20133 Milan, Italy; 3 Department of Experimental and Clinical Medicine, University of Florence, 50134 Florence, Italy; 4 Biophotonics@Tyndall, IPIC, Tyndall National Institute, Cork, T12R5CP Ireland

**Keywords:** Near-infrared spectroscopy, Laboratory techniques and procedures, Diagnostic markers, Respiratory tract diseases

## Abstract

The in vivo diagnosis and monitoring of pulmonary disorders (caused for example by emphysema, Covid-19, immature lung tissue in infants) could be effectively supported by the non-invasive sensing of the lung through light. With this purpose, we investigated the feasibility of probing the lung by means of time-resolved diffuse optics, leveraging the increased depth (a few centimeters) attained by photons collected after prolonged propagation time (a few nanoseconds). We present an initial study that includes measurements performed on 5 healthy volunteers during a breathing protocol, using a time-resolved broadband diffuse optical spectroscopy system. Those measurements were carried out across the spectral range of 600–1100 nm at a source-detector distance of 3 cm, and at 820 nm over a longer distance (7–9 cm). The preliminary analysis of the in vivo data with a simplified homogeneous model revealed a maximum probing depth of 2.6–3.9 cm, suitable for reaching the lung. Furthermore, we observed variations in signal associated with respiration, particularly evident at long photon propagation times. However, challenges stemming from both intra- and inter-subject variability, along with inconsistencies potentially arising from conflicting scattering and absorption effects on the collected signal, hindered a clear interpretation. Aspects that require further investigation for a more comprehensive understanding are outlined.

## Introduction

The lungs can be regarded as a mixture of air and tissue with a water-equivalent density. Accordingly, they can be visualized non-invasively through the attenuation of x-rays from an external source. In clinical practice, both conventional chest radiography (CXR) and computed tomography (CT) are widely used to detect pulmonary abnormalities. Thanks to its inherent tomographic properties, CT is superior to conventional CXR in visualizing the fine structure of the lung. Modern CT scanners can detect small solid lesions down to a size of 5 mm in width. However, neither CXR nor CT provide any direct information as regards the chemical composition of an abnormal lung density. The same limitation applies to thoracic ultrasound (US), which is increasingly used by the clinicians to evaluate pleural effusions or subpleural consolidations at bedside.

Assessing the lung composition and density can be useful to diagnose or monitor patients with emphysema, a pathologic condition characterized by an increased fraction of air per unit of lung volume. In patients with left heart failure, lung density may increase substantially due to the accumulation of water in the extravascular space of the lung (pulmonary edema). Also, lung inflammation with fluid accumulation (e.g., caused by infections, including Covid-19) leads to changes in lung density and composition. Lung density can be equally increased by an abnormal deposition of collagen in the lung interstitium (fibrosis).

Time Domain Diffuse Optical Spectroscopy (TD-DOS) is a photonic technique that permits to derive information on the characteristics of a biological tissue through the absorption ($\mu _a$) and reduced scattering ($\mu _s'$) coefficients via the study of photon propagation of short (ps) laser pulses within the medium^1,2^. So far, TD-DOS had been successfully applied to characterize non-invasively the composition of tissues or the function of organs, like the brain^3^ or the breast^4^, which are in close proximity to the surface of the body or can be assessed in transmittance geometry.

From the optical standpoint, the lungs are accessible over a large portion of the anterior and lateral chest wall. However, the varying thickness of the chest wall (on average 4 cm on the anterior—second rib—and 4.5 cm on the lateral side—fifth rib according to literature^5^) and its heterogeneous composition (skin, fat, and muscular tissue) pose a major challenge when using TD-DOS transcutaneously. In addition, the varying dimensions of the alveoli and distal conducting airways over the respiratory cycle may significantly affect the scattering of photons, making the interpretation of experimental data especially complex^6^.

As of now, optical techniques have been applied mostly for the diagnosis of lung cancer through invasive (bronchoscopy), or minimally invasive (needle biopsy) techniques^7^. They include diffuse reflectance spectroscopy^8,9^, endogenous^10^ or exogenous fluorescence spectroscopy^11,12^, optical coherent tomography^13^, and Raman spectroscopy^14^. Much effort is also being devoted to model the photon propagation through the lung by means of simulations^15,16^, anthropomorphic lung phantoms^17,18^, and ex vivo bovine lung tissues^19^. Conversely, there have not been many attempts to probe the lung non-invasively by optical transcutaneous means. In a first clinical study on 3 full-term babies, Gas in Scattering Media Absorption Spectroscopy (GASMAS) was used for non-invasive monitoring of water vapor in the lungs^20^. A larger study on 29 newborn infants demonstrated transcutaneous detection of oxygen in the lungs using GASMAS^21^. This approach was further validated also on a piglet animal model^22^. Yet, to the best of our knowledge, so far no non-invasive optical approach has been applied in vivo on the lungs in adults.

In the present study, we investigated the feasibility to sense the lung transcutaneously by simulations—instructed by in vivo spectroscopy measurements on the thorax—and by performing TD-DOS in healthy volunteers who were examined at suspended full inspiration and suspended full expiration.

## Materials and methods

### System set-up

Two TD-DOS systems were used for in vivo measurements on volunteers, namely a broadband spectrometer and a highly sensitive single-wavelength setup.

The broadband spectrometer was a laboratory workstation exploiting supercontinuum generation to automatically perform time domain measurements of the Distribution of Time-of-Flight (DTOF) over a wide spectral range, from 500 up to 1700 nm encompassing different detectors^23^. In the actual embodiment, an 80 MHz, 10 ps pulsed supercontinuum spectrum (SuperK EXTREME, NKT Photonics, Denmark) was sliced using a rotating Pellin-Broca prism followed by a 50 $\mu$m-core graded index fiber acting as spectral selection. Light was injected to and collected from the tissue using 1 mm-core step index fibers coupled to a custom-made Silicon Photomultiplier (SiPM) module^24^ connected to a Time-Correlated Single-Photon Counting (TCSPC) board (SPC130, Becker & Hickl GmbH, Germany). A variable optical attenuator, set on the source path, controlled the photon counting rate within the 1% single-photon statistics. A complete spectral acquisition from 600 nm to 1100 nm in steps of 10 nm with 4 s acquisitions required around 5 min.

The single-wavelength setup was optimised for maximal light harvesting. It was based on a prototypal 40 MHz, 25 ps high-power Four-Wave Mixing laser (Fianium Ltd, UK). The tissue was illuminated at 820 nm by a maximum power of 100 mW expanded to comply with the maximum permissible skin exposure. Re-emitted light was harvested using a 1 mm-core step index fiber coupled to a hybrid photomultiplier (HPM-100-50, Becker & Hickl GmbH, Germany), and connected with a TCSPC board (SPC130, Becker & Hickl GmbH, Germany).

### Simulations

To simulate the DTOFs in a layered tissue, a time-resolved GPU-accelerated Monte Carlo code^25^ was implemented making use of the microscopic Lambert-Beer approach^26^: for each detected photon, the path-length spent in each layer was saved. The simulation was run without absorption, weighting each trajectory afterwards, depending on the desired absorption in each layer. A total of 10$^{6}$ photons reaching the detector was collected for each simulation. The simulations were performed on a 10-core Dual Intel Xeon equipped with an Nvidia GeForce RtX3080Ti GPU board. The simulation time, mainly depending on the scattering and the source-detector separation, ranges from 5 to 4 min.

### In vivo protocol

Five healthy volunteers were recruited for the study, after approval from the institutional Ethical Review Board of Politecnico di Milano and after signing written informed consent. All experiments were performed in accordance with the Declaration of Helsinki and relevant guidelines and regulations. Demographic data are reported in Table 1.

For the broadband study, the subjects were lying supine breathing normally and with the probe positioned on the thorax in the upper-right region using a source-detector distance $\rho$ = 3 cm. Such a small distance does not require the use of a flexible probe to adapt to the curvature of the chest. The probe was therefore made of a small ($\sim$ 5 cm long, $\sim$ 2 cm wide, $\sim$ 1 cm thick) black polyvinyl chloride (PVC) plate with two holes permitting the insertion of both the source and the detection optical fibers at the proper distance. The surface of the PVC plate in contact with the skin of the subject was covered by a thin ($\sim$ 3 mm) layer of black adhesive neoprene tape, which grants both a reliable contact with the tissue thanks to its softness, and excellent light shielding, thus preventing the presence of direct light paths between the source and the detector fibers. The probe was hand-held by the subject, in tight contact with the chest. The absence of any detachment is proven by the absence of distortions in the DTOFs shapes as the the TD operation allows for reliable monitoring of the probe contact. Indeed, any slight detachment would result into a highly distorted rising edge of the DTOF acquired at the time of detachment, since direct light paths would result into photon detections occurring earlier than most of the photons travelling inside the chest.

For the single-wavelength study, the subject was lying supine, with the probe set in two locations on the thorax, namely on the anterior surface of the right hemithorax between the second (UR) and fifth (DR) intercostal space along the mid clavicular line. The source-detector distance $\rho$ was set to the maximum value ensuring a count-rate around 1 Mcounts/s, resulting in $\rho$ = $7 - 9$ cm depending on the subject and location. This large distance implies the use of a flexible probe to adapt to the curvature of the chest. The probe was therefore made of a large ($\sim$ 12 cm long, $\sim$ 6 cm wide, $\sim$ 2 cm thick) black neoprene plate with holes permitting the insertion of both the source and the detection optical fibers at the chosen distance. Similarly to the case of the broadband study, neoprene grants both a reliable contact with the tissue and excellent light shielding properties. In this case, the two opposite sides of the probe were connected to two elastic rubber bands fastened to two Velcro stripes at their ends. The probe was secured to the subject’s thorax by connecting the Velcro stripes on the opposite side of the torso. Also in this case the absence of detachments was verified in post-processing thanks to the absence of distortions in the DTOF shapes. The subjects were asked to follow a suspended full inspiration and suspended full expiration at a given pace to provide two intervals with the lung inflated (IN) or deflated (OUT). Two protocols were applied on each subject with 5 repetitions of the two phases lasting 10 s each (Prot10) or 10 repetitions with 5 s for each phase (Prot5). Only for Prot5 the first repetition was performed at normal breathing to get a reference state. Each protocol was repeated twice in each location, resulting in 8 complete series of acquisitions per subject. The application of the two protocols enables on the one hand (Prot10) to have a fairly large plateau to calculate differences in the IN and OUT phase, and on the other hand (Prot5) to verify that observed changes with respiration were not due to prolonged apnea. Finally, they serve as an indirect proof of reproducibility of the measurements.

### Data analysis

The DTOFs obtained with the spectral measurements were analyzed using a homogeneous solution of the Diffusion Equation under the extrapolated boundary conditions using $\mu _a$ and $\mu _s'$ as free fitting parameters. The Instrument Response Function (IRF) obtained facing the injection and collection fibers was convoluted with the theoretical model. The fitting range included points with a number of counts >80% and >1% of the peak value on the leading and falling edge of the DTOF, respectively. Tissue composition was derived from the retrieved absorption applying the Beer-Lambert law, that is interpreting the overall tissue absorption (at each wavelength) as the linear combination of the contributions from 5 key tissue absorbers, namely oxy- (Hb0$_2$), and deoxy-hemoglobin (Hb), water, lipids, and collagen. Reduced scattering spectra were best fitted with an empirical power law^27^:

1 $$\begin{aligned} \mu _s'(\lambda )=a(\lambda /\lambda _0)^{-b} \end{aligned}$$

where $\lambda _0 = 600$ nm, the scattering amplitude *a* provides information on the density of scattering centers, and the scattering power *b* on their size.

The DTOFs obtained with the single-wavelength measurements were analyzed using a fit with the homogeneous model similarly to the spectral measurements. For better visualization, a folding average over the 5 (10) repetitions of the protocol was applied. Further, the gated intensity was calculated for different time-windows of the DTOFs. The relative contrast of the gated reflectance signal R(t) with respect to a reference state R$_0$(t) was derived as:

2 $$\begin{aligned} C(t)=\frac{\int _{t}^{t+\Delta t} R(t')dt' - \int _{t}^{t+\Delta t} R_0(t')dt'}{\int _{t}^{t+\Delta t} R_0(t')dt'} \end{aligned}$$

where $t'$ is the photon arrival time, while *t* and $\Delta t$ are the starting edge and the width of the gated temporal window. For the *in vivo* protocols, $R_0$ is assumed to be the average of *R* over the whole exercise, therefore *C* represents the relative change in gated signal with respect to the mean value.

Equation 2 was used also to calculate the relative contrast for the Monte Carlo simulations. In that case, we assumed the unperturbed state as $R_0$.

## Results and discussion

### Diffuse optical spectroscopy of the human chest

**Figure 1:**
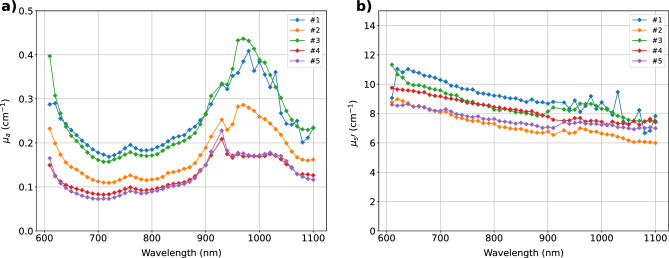
Absorption (**a**) and reduced scattering (**b**) spectra on the chest of 5 healthy volunteers using a source-detector distance $\rho = 3$ cm and a homogeneous model.

As a first step to estimate the optical properties of the tissues overlaying the lung, we measured the absorption and reduced scattering spectra on the chest region of healthy volunteers using an intermediate source-detector distance $\rho$ = 3 cm. Fig. 1 displays the absorption (panel (a)) and reduced scattering spectra (panel (b)) of 5 subjects (see Table 1 for demographics) obtained assuming a homogeneous model. The clear absorption peak around 975 nm, ascribed to water, is typically observed in muscle tissue, and it becomes dominant, hiding the lipid peak at 930 nm, when moving from subject #5 to #1. Differences in the region <800 nm are mostly due to blood content, which again contributes stronger to the absorption of muscle tissue. Overall, around 800 nm, where we performed large-$\rho$ measurements ($\rho =7-9$ cm), $\mu _a \thickapprox 0.1 - 0.2$ cm$^{-1}$ and $\mu _s' \thickapprox 7 - 9$ cm$^{-1}$. Table 1 reports the average tissue composition obtained from the Beer-Lambert law assuming 5 absorbers (Hb, HbO$_2$, water, lipids, and collagen) and the empirical power law dependence for the scattering spectrum. The mean tissue composition of different subjects confirms the attributions on fat or muscle predominance made from the visual analysis of the absorption spectra.

**Figure 2:**
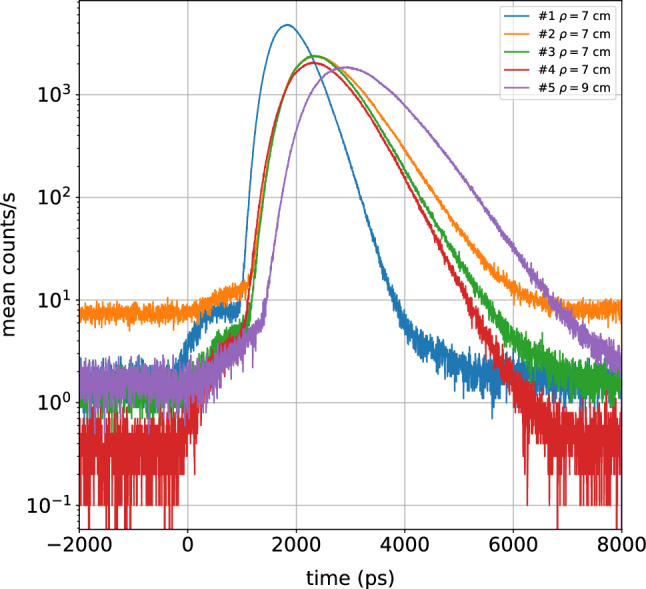
Distributions of photon times of flight (DTOFs) for the 5 volunteers on the chest in reflectance geometry using the largest source-detector distance ($\rho =7 - 9$ cm) yielding a full count-rate collection statistics of $\approx 1$ Mcounts/s. The maximum photon travelling time is up to $4 - 8$ ns depending on the subject. The instant $t=0$ corresponds to the peak position of the IRF.

To get a first guess on the maximum detectable photon travelling time, which determines the maximum explored depth in the medium^28,29^, in Fig. 2 we show the DTOFs measured on the chest of the same 5 subjects using quite a large $\rho$ = $7 - 9$ cm. We worked at a single wavelength (820 nm), selecting the maximum acceptable $\rho$ value, that is the one which leads the max count rate acceptable for correct operation of the instrumentation (1 Mcounts/s, due to saturation thresholds of the electronics and to the requirements for single-photon regime). Increasing $\rho$ beyond that limit would simply decrease the number of collected photons at any times^28^. As expected, the temporal profile is quite broad and extends to late times, with early photons arriving not earlier than 1 ns after the pulse injection time (*t* = 0 ns). The maximum photon arrival time, or better the maximum *t* when the DTOF stands out of the noise, ranges from 4 ns for subject #1 up to 8 ns for subject #5. These values can provide a first hint to interpret the simulations presented below, and to guess the maximum investigation depth of the measurements. The absorption and scattering properties derived using a homogeneous model for this large $\rho$ at 820 nm are also reported in Table 1.

**Table 1:** Subjects’ demographics, mean tissue composition and scattering parameters obtained at $\rho$ = 3 cm, and optical properties at 820 nm obtained with longer source-detector distance ($\rho = 7-9$ cm).

Subject	Age	BMI	Upper layer							Lower layer	
			Hb	HbO$_2$	Water	Lipid	Collagen	a	b	$\mu _a$	$\mu _s'$
	(y)	(kg/m$$^2$$)	$$(\mu M)$$	$$(\mu M)$$	(g/cm$$^3)$$	(g/cm$$^3)$$	(g/cm$$^3)$$	(cm$$^{-1})$$		(cm$$^{-1})$$	(cm$$^{-1})$$
#1	32	21.8	1.5	25.1	0.34	0.43	0.21	11.1	0.63	0.32	11.7
#2	65	23.1	3.0	15.5	0.29	0.63	0.11	9.0	0.78	0.14	7.3
#3	52	19.1	6.9	26.4	0.49	0.67	0.14	10.5	0.75	0.34	11.6
#4	34	24.1	0.8	11.4	0.12	0.69	0.04	10.0	0.59	0.15	8.2
#5	49	25.8	1.3	15.8	0.14	0.85	0.04	9.0	0.61	0.09	5.3

### Simulations

**Figure 3:**
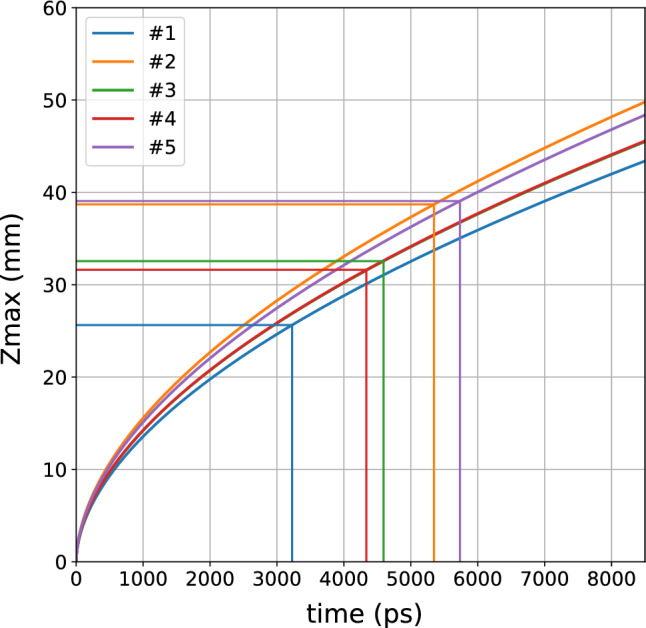
Mean value of the maximum depth of photon trajectories ($z_{max}$) as a function of photon arrival time derived for the $\mu _s'$ properties of the 5 subjects. The straight lines indicate the maximum *t* yielding a count-rate >10,000 counts/s over a late gate for data in Fig. 2, and the corresponding value of $z_{max}$. The green curve (subject #3) is hidden by the red one (subject #4)), as they are very close due to the markedly similar scattering coefficients at 820 nm.

As an initial step to answer the question of whether it is possible to reach the lung, we estimated the maximum depth reached by photons in a homogeneous diffusive medium in reflectance geometry. To that purpose, we adopted the approach proposed in Ref.^29^, deriving the mean value of the maximum depth $z_{max}$ of all possible photon trajectories in a semi-infinite medium (see Material and Methods for details). Fig. 3 displays the resulting $z_{max}$ as a function of photon travelling time *t* for the $\mu _s'$ derived for each subject at 820 nm with $\rho$ = 3 cm (Fig. 1), which reasonably represents the mean scattering properties in the superficial chest layers that need to be traversed to reach the lung. For a homogeneous medium, once fixed $\mu _s'$, the mean depth is rigorously independent of both $\mu _a$ and $\rho$, which affect the number of collected photons but do not alter the depth distribution of photon trajectories for a given *t*. Calculating the maximum photon arrival time $t_{max}$ yielding >10,000 counts/s for a late gate (shot noise = 1%), we obtain $t_{max} \approx 3.2-5.7$ ns, which leads to $z_{max} \approx 2.6-3.9$ cm for the 5 subjects, as represented in Fig. 3.

**Figure 4:**
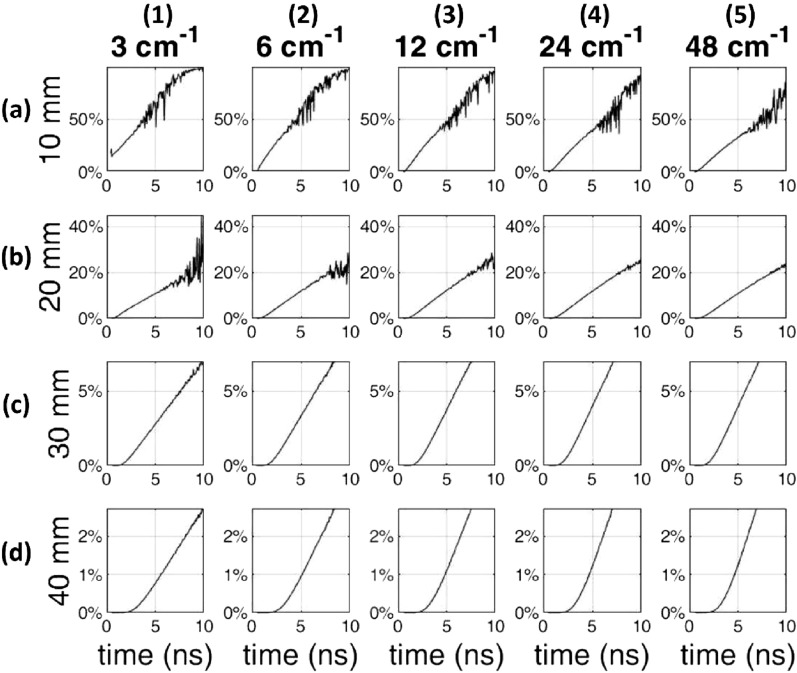
Monte Carlo simulations of the relative contrast *C* as a function of photon arrival time *t* for an absorption reduction of 10% in the lung absorption for different values of $\mu _s'$ assumed homogeneous (columns) and of the lung depth (rows). The source-detector distance is 6 cm.

As a second step, for a more realistic scenario, we assumed a two-layer medium composed of the lung (bottom layer) and all the overlaying tissues (top layer). Using a Monte Carlo code, we simulated a 10% reduction in lung absorption to mimic the reduction in lung density due to inhalation in our measurements and a pathologic condition in future diagnostic measures^30^. Fig. 4 shows the relative contrast *C* between the perturbed and unperturbed states plotted as a function of the photon arrival time *t* for different depths of the lung layer $z_{lung}$ (rows, based on the values of the previous step) and for different choices of $\mu _s'$ in the lung (columns), while assuming $\mu _s'=7$ cm$^{-1}$ for the upper layer, corresponding to an average value of the scattering properties obtained as a function of wavelength on the 5 subjects (Fig. 1). Relative contrast $C \approx 2-3\%$ is achieved down to $z_{lung} \thickapprox$ 3 cm for *t* = 4 ns, and even down to $z_{lung} \thickapprox$ 4 cm for *t* = 8 ns. In the literature there is not yet a sound knowledge of the lung scattering properties, and we opted to span a wide range of lung scattering properties. Quite surprisingly, the lung scattering does not significantly affect *C*. This result relaxes the threat that an extremely high $\mu _s'$ in the lung could completely prevent lung sensing. Yet this agrees with the previous argument that the photon travelling depth is mostly influenced by the upper layer. In a bilayered medium, the scattering of the upper layer could thus be an additional variable to investigate (for example through short source-detector distance measurements) and to take into account, although we observed limited inter-subject variability (see Fig. 1 and Table 1).

### In vivo breathing protocol

As an in vivo trial of the actual chances to sense the lung, we performed in vivo measurements at 820 nm on the 5 subjects following the breathing protocol described previously. The aim of this protocol was to produce a decrease in both $\mu _a$ and $\mu _s'$, of the lung during the inhalation phase, due to the reduction of lung density, and check the sensitivity of the optical measurement to such changes.

**Figure 5:**
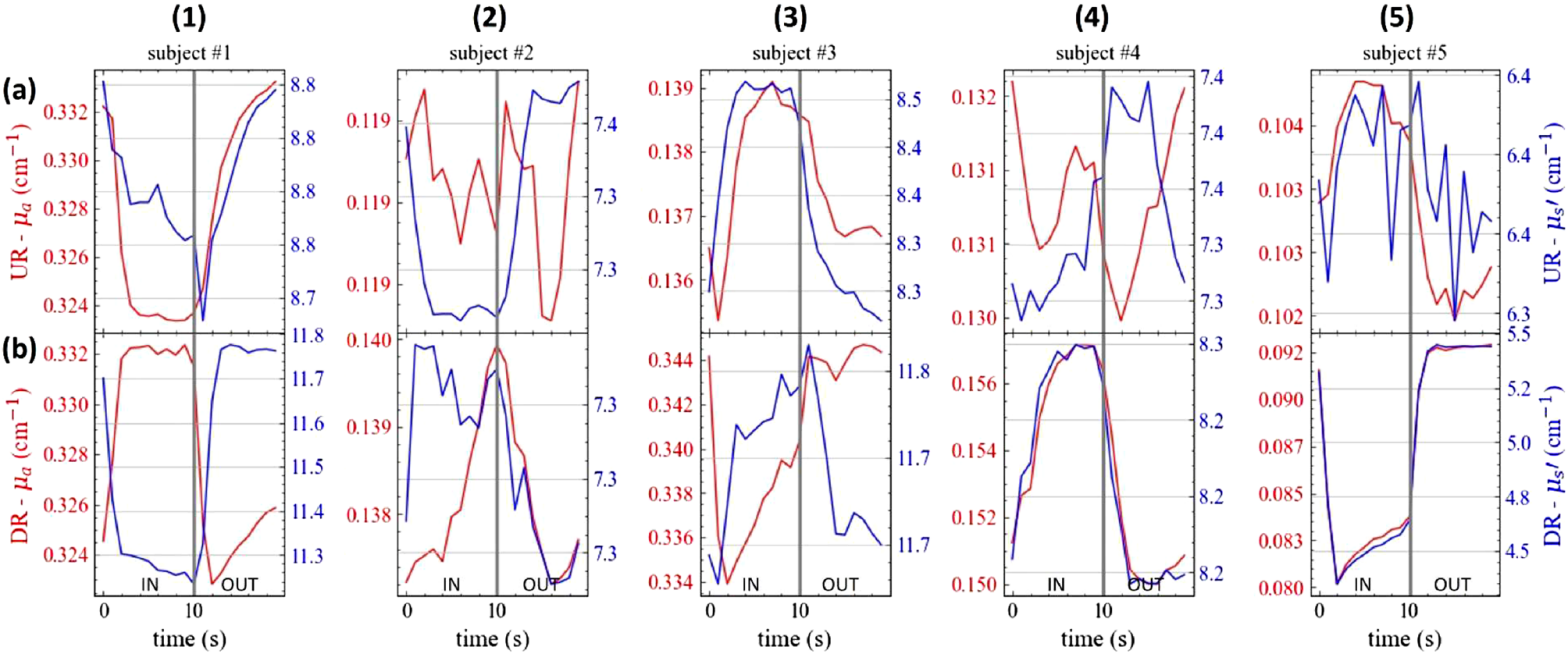
In vivo time evolution of the absorption (red) and reduced scattering (blue) coefficients at 820 nm during the inhalation protocol Prot10 for the 5 volunteers (columns, numbers) and 2 locations (rows, letters) over the lung. At $t=0$ s, the subject is asked to inhale, while at $t=10$ s the subject starts to exhale. Values refer to the folding average.

**Figure 6:**
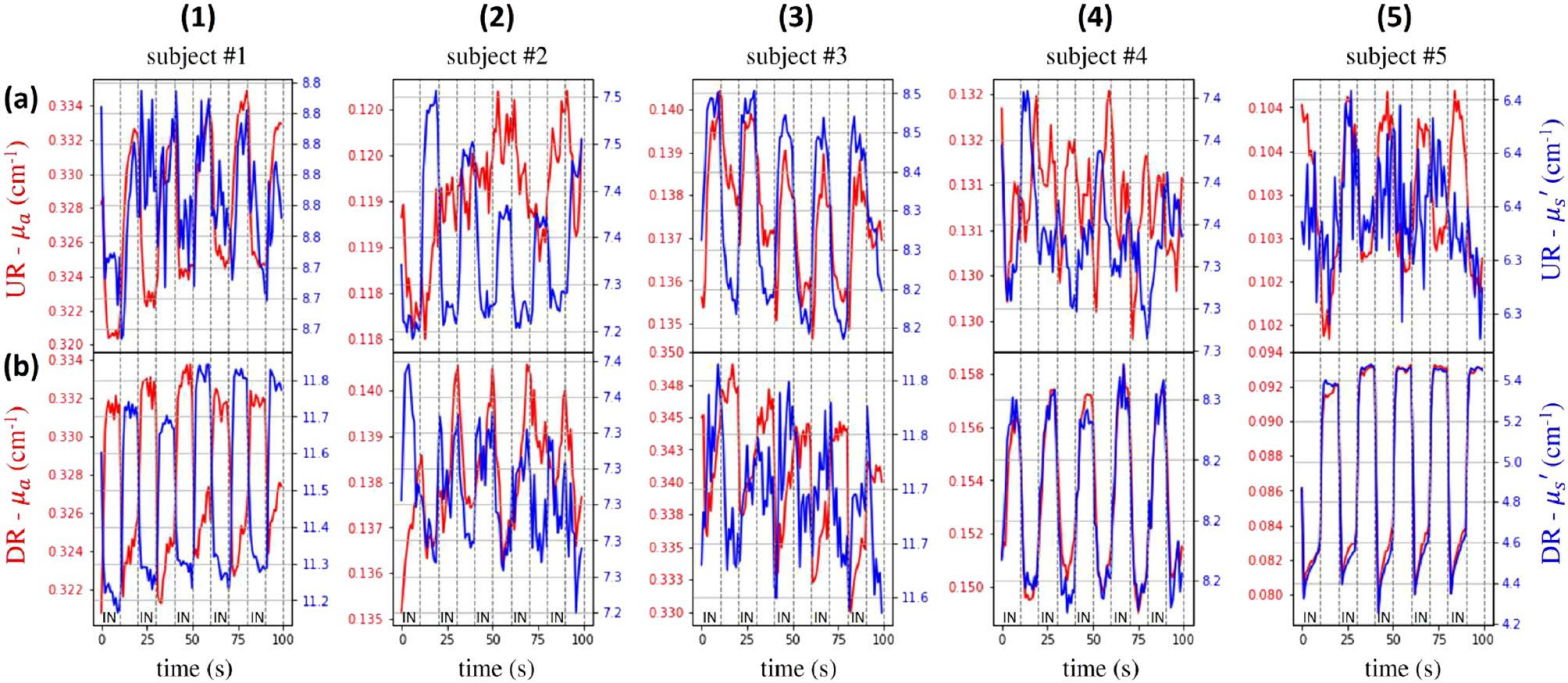
Non-refolded in vivo time evolution of the absorption (red) and reduced scattering (blue) coefficients at 820 nm during the inhalation protocol Prot10 for the 5 volunteers (columns, numbers) and 2 locations (rows, letters) over the lung. At t = 0 s, the subject is asked to inhale (IN), while at t = 10 s the subject starts to exhale. The processed is repeated 5 times.

Fig. 5 reports the evolution of $\mu _a$ (red, left axis) and $\mu _s'$ (blue, right axis), as obtained using a homogeneous fit over the time of the task for the 5 subjects (columns) and 2 positions on the chest, namely an up-right location (UR, top row) and a down-right location (DR, bottom row). The protocol in use was Prot10 and the folding average was applied over the exercise time. In general, there is a task-related change in the fitted optical properties. Yet, the results are somehow contradictory with large inter- and intra-subject differences. In some cases, (e.g., #5-DR), both $\mu _a$ and $\mu _s'$, decrease with inhalation as expected from the reduction in lung density, but in other cases the behavior is just the opposite (e.g., #3-UR), or even a parameter increases while the other decreases (e.g. #1-DR). Also intra-subject differences among the two positions are observed (e.g., #1). The task-related alteration is observed also in the not-refolded time evolution (Fig. 6), yet again with quite different patterns. According to our own experience in TD-DOS, a homogeneous fit of a large-$\rho$ measurement typically enhances the optical properties of the deeper (few cm depth) layer^31^. This is particularly true for $\mu _a$ while information on $\mu _s'$, comes from the superficial layer^31^.

**Figure 7:**
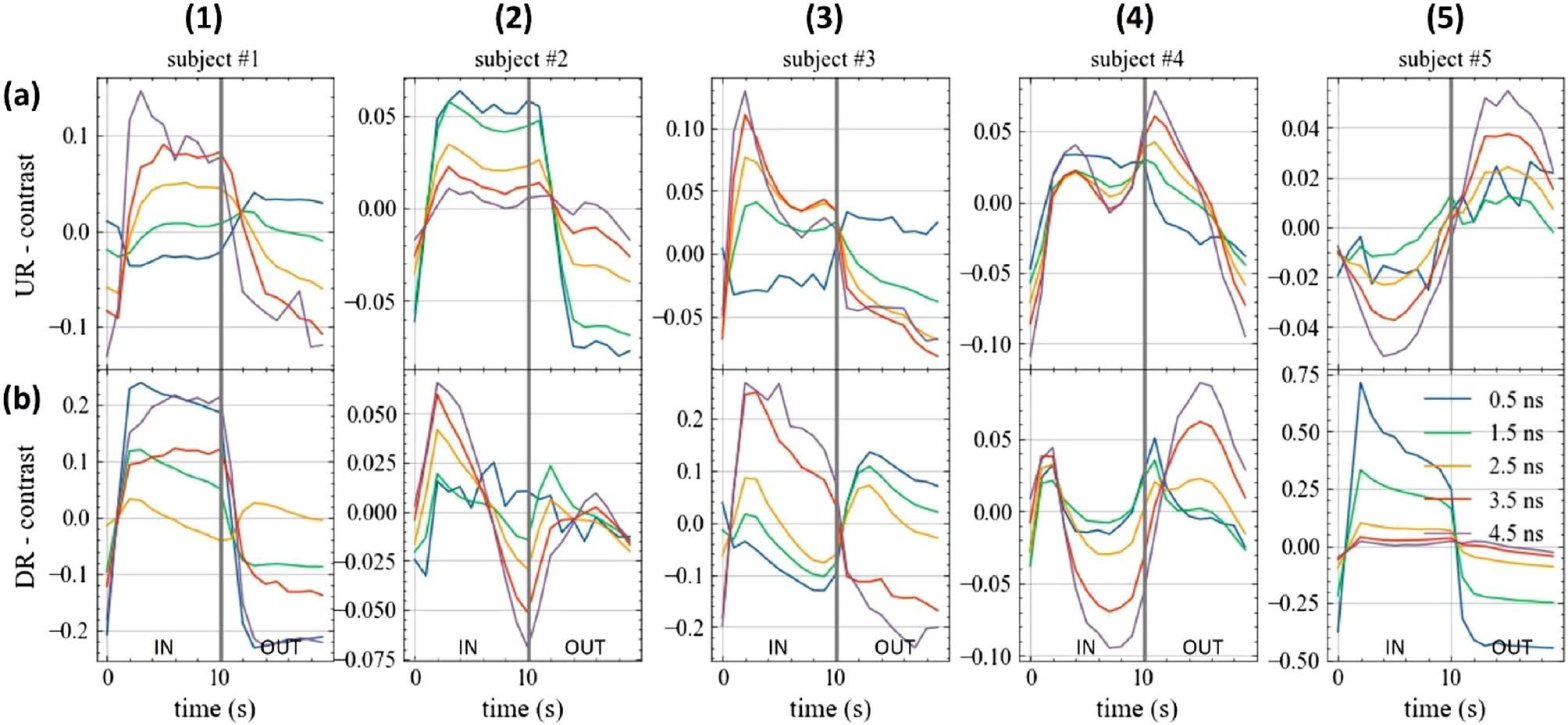
In vivo time evolution of the relative contrast for different time gates (see legend) at 820 nm during the breathing protocol Prot10 for the 5 volunteers (columns, numbers) and 2 locations (rows, letters) over the lung. At $t=0$ s, the subject is asked to inhale, while at $t=10$ s the subject starts to exhale. Values refer to the folding average.

**Figure 8:**
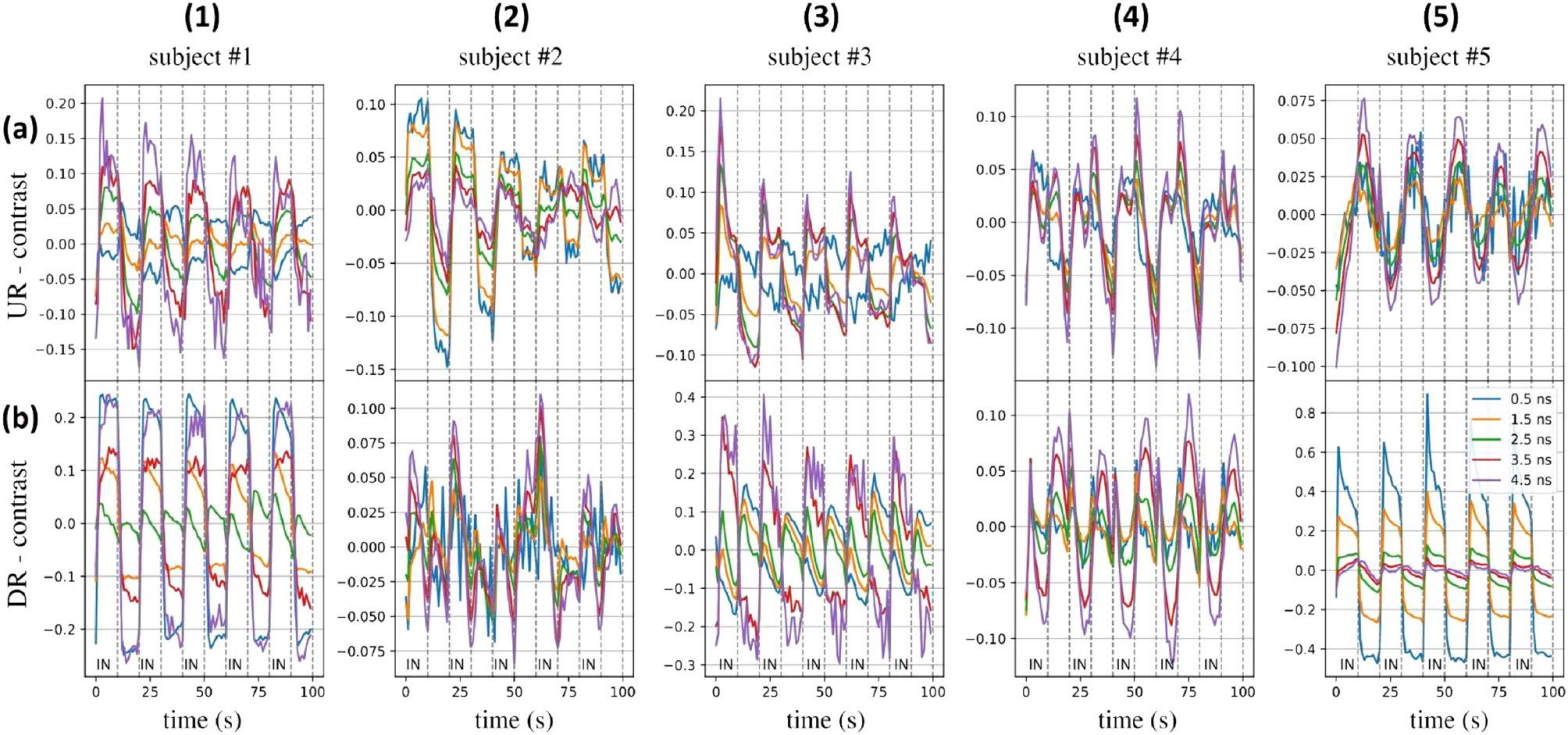
Non-refolded in vivo time evolution of the relative contrast for different time gates (see legend) at 820 nm during the breathing protocol Prot10 for the 5 volunteers (columns) and 2 locations (rows) over the lung. At t = 0 s, the subject is asked to inhale (IN), while at t = 10 s the subject starts to exhale. The process is repeated 5 times.

The other protocol (Prot5) shows substantially similar results (see Supplementary Fig. S1 and Fig. S2 for the refolded and not-folded representation, respectively). As a different view on the same data, we analysed the relative contrast *C* in photon counts for different time gates over the IN and OUT phases, respectively. Fig. 7 displays *C*(*t*) for 5 different gates, with *t* ranging from 0.5 to 4.5 ns and $\Delta t$ = 0.5 ns as a function of the refolded time for the 5 subjects (columns) and the 2 positions (rows). The figure shows data from protocol Prot10 with folding average. In all 10 plots, apart from #5-UR and possibly #4-DR, we observe a reduction in *C* for late gates, that is a reduction in signal intensity when the lung tissue gets denser (OUT phase). Fig. 8 shows the corresponding not-folded results. The other protocol (Prot5) shows substantially similar results (see Supplementary Fig. S3 and Fig. S4 for the refolded and not-folded visualization of Prot5, respectively).

**Figure 9:**
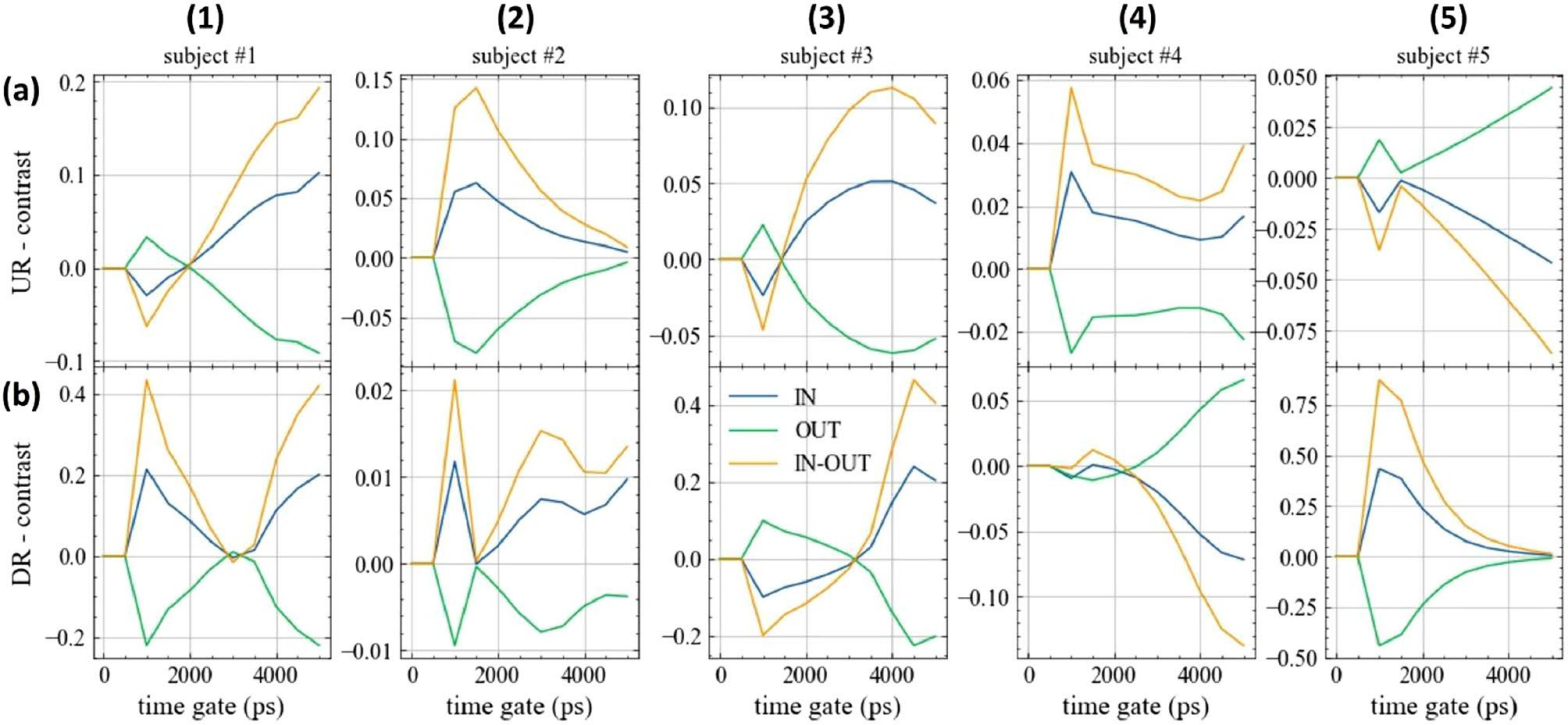
Dependence of the relative contrast *C* *vs* photon propagation time during the plateau of the inhale (IN) or exhale (OUT) phases, together with the difference (IN-OUT) for the 5 volunteers (columns, numbers) and 2 locations (rows, letters) for Prot10. In most cases, *C* (i.e. the collected signal) increases from the OUT to the IN phase, yet with strong inter- and intra-subject variability and complex evolution for increasing photon propagation time.

To better visualize the signal perturbation as a function of the photon arrival time *t* we calculated the average contrast between the plateau state of the inspiration and expiration phases (breath hold). Fig. 9 shows the mean value of *C*(*t*) during the IN phase (blue line), the OUT phase (green line) and the IN-OUT difference (orange line) as a function of the gate time *t*. Columns refer to the subjects, while rows to the position. As expected, the IN-OUT line is positive for late gates for most subjects. Yet, following simulations in Fig. 4, *C*(*t*) should increase monotonically with *t* due to the increase in mean probed depth and therefore in lung contribution. The situation depicted in Fig. 9 is more complex. There seem to be two opposing factors causing the IN-OUT curve to cross the null contrast at a given *t* and then to diverge for larger *t* values. In most cases, *C* is higher in the OUT than in the IN phase, yet with strong inter- and intra-subject variability and complex evolution for increasing photon propagation time. Similar results are obtained for the Prot5 protocol (see Supplementary Fig. S5).

All these in vivo results seem to point out that we are likely reaching the lung, but competing factors make the interpretation of the breathing protocol not straightforward. In the following, we analyze five issues to be considered.

**Figure 10:**
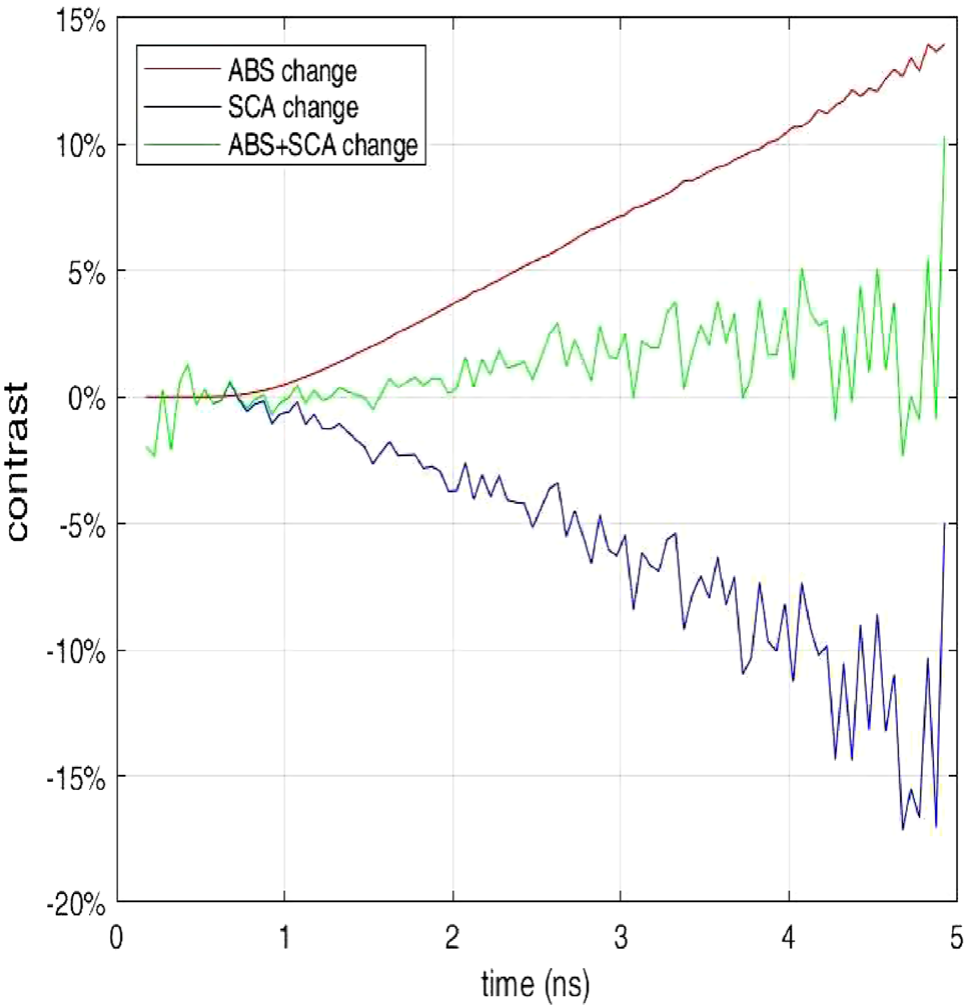
Monte Carlo simulation of the relative contrast as a function of the photon arrival time (*t*) assuming an increase in lung air content leading to a 20% reduction only in $\mu _a$ (ABS) in $\mu _s'$ (SCA) or rather the combined reduction of both (ABS+SCA).

The first factor is the interplay of $\mu _a$ and $\mu _s'$, in the lung during inhalation. Both should decrease due to the lower tissue density. Yet, a linear dependence of the absorption on tissue density (and constituent concentrations) is expected, while the scattering reduction is not necessarily linear with tissue density due to the contribution of air-filled alveoli acting as independent scattering centers^32^. Furthermore, complex phenomena such as anomalous diffusion due to the air gaps might play a role^33^. As a very simple case, still based on the hypothesis of linear behavior of both absorption and scattering, in Fig. 10 we plot the Monte Carlo simulation of the contrast *C*(*t*) assuming an identical reduction by 20% of $\mu _a$ and $\mu _s'$, in the lung. A reduction only in absorption (red line) yields a linear increase of *C* vs *t*. A reduction only in scattering (blue line) yields a linear decrease of *C* vs *t* of a comparable amount. The combined effect of both parameters (green line) dramatically reduces the overall contrast.

A second factor resides in the lung scattering, which could be extremely high, thus hampering lung exploration. There is still poor knowledge on the lung optical properties, particularly in vivo. Some ex vivo studies report values for $\mu _s'$ up to 20 cm$^{-1}$ at 800 nm (calculated using Eq. 1 and tabulated *a* and *b* values)^34^, but such a high value could be due to the room temperature adopted in measurements leading to overestimation of $\mu _s'$ with respect to the in vivo conditions. In any case, simulations presented in Fig. 4 indicate that the scattering of the lower layer (lung) is not so critical in determining the sensitivity to lung properties beyond a given photon travelling time over a large range of lung $\mu _s'$ values.

A third factor to be considered is the pleura, which is the two-foil membrane enveloping lungs and chest wall, separated by a lubrication fluid, which permits smooth sliding and expansion of the lung during the inhalation act. From the optical point of view, the pleura could cause a light-guiding effect, creating contamination from more superficial photons even at large time values, thus hiding deep photon signals. Similarly, in the brain the cerebrospinal fluid (CSF) was questioned to cause light-guiding effects and artefacts in functional Near-Infrared Spectroscopy (fNIRS). This issue was long discussed in the initial phases of fNIRS with opposite conclusions, but the question was then settled by strong evidence of cortex-related activation in many studies and agreement with fMRI scans^35^. The pleura thickness - extrapolated from animal data - should be in the $30-40\;\mu m$ range^36^, so definitely lower than the CSF layer.

A fourth aspect is the inadequacy of the simple analysis tools used in this study to describe the complex chest structure. The homogeneous fit proposed in Fig. 5 is a clear oversimplification of the actual geometry. Yet, in previous works, confirmed with phantom experiments, we demonstrated that $\mu _a$ spectra obtained for reasonable large *t* values using a homogeneous fit tend to adhere to the lower (few cm deep) layer^31^. The photon pathlength in the upper layer quickly reaches a stable plateau after a given *t* while the pathlength in the lower layer linearly increases with *t* above the same threshold. Thus, the fit on $\mu _a$ is mainly affected by the lower layer properties being related to the temporal slope on the tail of the DTOF. Still, this simple argument does not hold true for $\mu _s'$, and in our case the variation in both $\mu _a$ and $\mu _s'$ might not be properly captured by this simple model.

A fifth concern is related to the changes in optical properties in the lung during the inhalation phase and the conversion of Hb to HbO$_2$. Still, the adopted wavelength (820 nm) is close to the isosbestic point for the absorption of Hb and HbO$_2$ and consequently the overall absorption should depend weakly on the oxygenation status. Also, in the long breath holding protocol (Prot10) the observed changes are quite rapid and possibly related to the inhalation act, and do not show a continuous increase with laboratory time as expected for a progressive uptake of O$_2$.

## Conclusion

We have presented the first study on the transdermal optical accessibility of the lung using time domain diffuse optics based on Monte Carlo simulations and in vivo measurements on 5 healthy volunteers. In vivo broadband absorption and reduced scattering spectra in the 600-1100 nm range at $\rho$ = 3 cm, analyzed using a homogeneous model, allowed the estimate of the range of optical properties of superficial tissues to be traversed to reach the lung. In particular, around 800 nm we obtained $\mu _a \thickapprox 0.1 - 0.2$ cm$^{-1}$ and $\mu _s' \thickapprox 7 - 9$ cm$^{-1}$. Even at larger $\rho$ ($7 - 9$ cm), the measured optical properties at 820 nm fall in a similar range. The same large $\rho$ in vivo measurements showed that a photon travelling time up to $t_{max} \approx 3.2-5.7$ ns with at least 10,000 counts/s (shot noise = 1%) in a late gate could be reached for different subjects.

Also, two-layer Monte Carlo simulations with different choices of lung $\mu _s'$ in a wide range of values ($3-48$ cm$^{-1}$) showed a relative contrast $C \approx 2-3\%$ for a 10% reduction in lung $\mu _s'$ down to a depth of 3 (4) cm for *t* = 4 (8) ns, which should be enough to probe the lungs. The in vivo measurements on a paced breathing protocol showed clear task-related changes in time domain signals. Yet, the analysis of DTOFs using a homogeneous model showed contradictory results, not in agreement with the expected decrease in both $\mu _a$ and $\mu _s'$, during inhalation. Conversely, the plot of the relative contrast *C* for the photon counts in temporal gates displayed a more consistent trend with a general increase in reflectance signal during the inhalation phase, as expected from diffuse optics models. Still, the contrast is not always increasing upon increasing photon arrival times, as foreseen by a two-layered model, likely due to the opposing effects of absorption and scattering.

In conclusion, we have not yet a sound evidence that we detected lung-related properties in vivo. Yet, we have provided some important pieces of information to guide further work: we estimated the optical properties of the chest wall, simulation scenarios were presented to help interpreting and guiding measures, and we designed and tested a potentially powerful protocol for in vivo validation. The next steps of our work will be devoted to: (1) the correlation between acquisitions and lungs’ movement and volume through a breath sensor; (2) the research and selection of the optimal wavelengths to enhance sensitivity to lungs’ composition with respect to the one of the overlying layer; (3) multi-position and multi-distance measurements to verify the effect of superficial heterogeneity; (4) the use of US images to better locate the probe and correlate measurement results with the anatomy of the investigated tissue; (5) the availability of open data to share experimental results. Furthermore, it would be interesting to compare results obtained with time domain measurements with what is achieved with a continuous-wave multi-distance approach.

Technology is advancing at a fast pace in TD-DOS, both in terms of reduction in cost-size and improvement in performances. In particular, the increase in area of new SiPMs detectors^37^ as well as their gating capabilities^38^ mean that larger photon travelling times and consequently penetration depths will be reached in the near future. What is not available yet is a clear understanding of the physics and physiology of chest diffuse optics and further studies are needed to get additional insight and in vivo validation.

### Supplementary Information

Supplementary Figures.

## Data Availability

The datasets generated and analysed during the current study are available from the corresponding author on reasonable request.

## References

[1] Durduran, T., Choe, R., Baker, W. B. & Yodh, A. G. Diffuse optics for tissue monitoring and tomography. *Rep. Prog. Phys.* **73**, 076701. 10.1088/0034-4885/73/7/076701 (2010). 26120204 10.1088/0034-4885/73/7/076701 PMC4482362

[2] Pifferi, A. *et al.* New frontiers in time-domain diffuse optics, a review. *J. Biomed. Opt.* **21**, 091310. 10.1117/1.JBO.21.9.091310 (2016). 27311627 10.1117/1.JBO.21.9.091310

[3] Torricelli, A. *et al.* Time domain functional NIRS imaging for human brain mapping. *NeuroImage* **85**, 28–50. 10.1016/j.neuroimage.2013.05.106 (2014). 23747285 10.1016/j.neuroimage.2013.05.106

[4] Grosenick, D., Rinneberg, H., Cubeddu, R. & Taroni, P. Review of optical breast imaging and spectroscopy. *J. Biomed. Opt.* **21**, 091311. 10.1117/1.JBO.21.9.091311 (2016). 27403837 10.1117/1.JBO.21.9.091311

[5] Schroeder, E. *et al.* Average chest wall thickness at two anatomic locations in trauma patients. *Injury* **44**, 1183–1185. 10.1016/j.injury.2013.03.027 (2013). 23618786 10.1016/j.injury.2013.03.027

[6] Durkee, M. S. *et al.* Light scattering by pulmonary alveoli and airway surface liquid using a concentric sphere model. *Opt. Lett.* **43**, 5001. 10.1364/OL.43.005001 (2018). 30320804 10.1364/OL.43.005001 PMC6528677

[7] Zeng, H., McWilliams, A. & Lam, S. Optical spectroscopy and imaging for early lung cancer detection: a review. *Photodiagn. Photodyn. Ther.* **1**, 111–122. 10.1016/S1572-1000(04)00042-0 (2004). 10.1016/S1572-1000(04)00042-0 25048182

[8] Spliethoff, J. W. *et al.* Improved identification of peripheral lung tumors by using diffuse reflectance and fluorescence spectroscopy. *Lung Cancer* **80**, 165–171. 10.1016/j.lungcan.2013.01.016 (2013). 23402823 10.1016/j.lungcan.2013.01.016

[9] Spliethoff, J. W. *et al.* Real-time in vivo tissue characterization with diffuse reflectance spectroscopy during transthoracic lung biopsy: A clinical feasibility study. *Clin. Cancer Res.* **22**, 357–365. 10.1158/1078-0432.CCR-15-0807 (2016). 26324737 10.1158/1078-0432.CCR-15-0807

[10] Lam, S., MacAulay, C., LeRiche, J. C. & Palcic, B. Detection and localization of early lung cancer by fluorescence bronchoscopy. *Cancer* **89**, 2468–2473. https://doi.org/10.1002/1097-0142(20001201)89:11+2468::aid-cncr253.3.co;2-m (2000). 10.1002/1097-0142(20001201)89:11+<2468::aid-cncr25>3.3.co;2-m 11147629

[11] Okusanya, O. T. *et al.* Intraoperative molecular imaging can identify lung adenocarcinomas during pulmonary resection. *J. Thorac. Cardiovasc. Surg.* **150**, 28-35.e1. 10.1016/j.jtcvs.2015.05.014 (2015). 26126457 10.1016/j.jtcvs.2015.05.014 PMC4828933

[12] Hernot, S., van Manen, L., Debie, P., Mieog, J. S. D. & Vahrmeijer, A. L. Latest developments in molecular tracers for fluorescence image-guided cancer surgery. *Lancet Oncol.* **20**, e354–e367. 10.1016/S1470-2045(19)30317-1 (2019). 31267970 10.1016/S1470-2045(19)30317-1

[13] Lam, S. *et al.* In vivo Optical Coherence Tomography Imaging of Preinvasive Bronchial Lesions. *Clin. Cancer Res.* **14**, 2006–2011. 10.1158/1078-0432.CCR-07-4418 (2008). 18381938 10.1158/1078-0432.CCR-07-4418 PMC2849640

[14] McGregor, H. C. *et al.* Real-time endoscopic Raman spectroscopy for in vivo early lung cancer detection. *J. Biophotonics* **10**, 98–110. 10.1002/jbio.201500204 (2017). 26748689 10.1002/jbio.201500204

[15] Sikorski, Z., Furmanczyk, M. & Przekwas, A. J. Modeling of photon migration in the human lung using a finite volume solver. In Oraevsky, A. A. & Wang, L. V. (eds.) *Photons Plus Ultrasound: Imaging and Sensing 2006: The Seventh Conference on Biomedical Thermoacoustics, Optoacoustics, and Acousto-optics*, vol. 6086, 60861Z. 10.1117/12.646329 (2006).

[16] Singh, R., Sasmal, A. & Mishra, S. C. Nanoparticle mediated transmittance signals from pulsed laser irradiated cancerous lung as a function of respiration. *Optik* **126**, 5605–5609. 10.1016/j.ijleo.2015.09.242 (2015). 10.1016/j.ijleo.2015.09.242

[17] Larsson, J. *et al.* Development of a 3-dimensional tissue lung phantom of a preterm infant for optical measurements of oxygen-Laser-detector position considerations. *J. Biophotonics* **11**, e201700097. 10.1002/jbio.201700097 (2018). 10.1002/jbio.201700097 28816029

[18] Pacheco, A., Li, H., Chakravarty, M., Sekar, S. K. V. & Andersson-Engels, S. Anthropomorphic optical phantom of the neonatal thorax: A key tool for pulmonary studies in preterm infants. *J. Biomed. Opt.* **25**, 1–11. 10.1117/1.JBO.25.11.115001 (2020). 10.1117/1.JBO.25.11.115001 PMC7670093 33205636

[19] Lacerenza, M. *et al.* Functional monitoring of lung tissue using a hybrid hyperspectral Time-Resolved GASMAS system: A systematic study on ex vivo sample. In *Biophotonics Congress: Biomedical Optics 2020 (Translational, Microscopy, OCT, OTS, BRAIN)*, vol. Part F179-, SW1D.2. 10.1364/OTS.2020.SW1D.2 (Optica Publishing Group, Washington, D.C., 2020).

[20] Lundin, P. *et al.* Noninvasive monitoring of gas in the lungs and intestines of newborn infants using diode lasers: Feasibility study. *J. Biomed. Opt.* **18**, 127005. 10.1117/1.JBO.18.12.127005 (2013). 24362929 10.1117/1.JBO.18.12.127005

[21] Svanberg, E. K. *et al.* Diode laser spectroscopy for noninvasive monitoring of oxygen in the lungs of newborn infants. *Pediatr. Res.* **79**, 621–628. 10.1038/pr.2015.267 (2016). 26679152 10.1038/pr.2015.267

[22] Svanberg, E. K. *et al.* Changes in pulmonary oxygen content are detectable with laser absorption spectroscopy: Proof of concept in newborn piglets. *Pediatr. Res.* **89**, 823–829. 10.1038/s41390-020-0971-x (2021). 32534453 10.1038/s41390-020-0971-x PMC7322222

[23] Sekar, S. K. V. *et al.* Diffuse optical characterization of collagen absorption from 500 to 1700 nm. *J. Biomed. Opt.* **22**, 015006. 10.1117/1.JBO.22.1.015006 (2017). 10.1117/1.JBO.22.1.015006 28138693

[24] Martinenghi, E. *et al.* Spectrally resolved single-photon timing of silicon photomultipliers for time-domain diffuse spectroscopy. *IEEE Photonics J.* **7**, 1–12. 10.1109/JPHOT.2015.2456070 (2015). 10.1109/JPHOT.2015.2456070

[25] Alerstam, E., Svensson, T. & Andersson-Engels, S. Parallel computing with graphics processing units for high-speed Monte Carlo simulation of photon migration. *J. Biomed. Opt.* **13**, 060504. 10.1117/1.3041496 (2008). 19123645 10.1117/1.3041496

[26] Sassaroli, A. & Martelli, F. Equivalence of four Monte Carlo methods for photon migration in turbid media. *J. Opt. Soc. Am. A* **29**, 2110. 10.1364/JOSAA.29.002110 (2012). 10.1364/JOSAA.29.002110 23201658

[27] Mourant, J. R., Fuselier, T., Boyer, J., Johnson, T. M. & Bigio, I. J. Predictions and measurements of scattering and absorption over broad wavelength ranges in tissue phantoms. *Appl. Opt.* **36**, 949. 10.1364/AO.36.000949 (1997). 18250760 10.1364/AO.36.000949

[28] Torricelli, A. *et al.* Time-resolved reflectance at null source-detector separation: Improving contrast and resolution in diffuse optical imaging. *Phys. Rev. Lett.* **95**, 078101. 10.1103/PhysRevLett.95.078101 (2005). 16196825 10.1103/PhysRevLett.95.078101

[29] Martelli, F. *et al.* There’s plenty of light at the bottom: Statistics of photon penetration depth in random media. *Sci. Rep.* **6**, 27057. 10.1038/srep27057 (2016). 27256988 10.1038/srep27057 PMC4891734

[30] Euliano, T. Y., Gravenstein, J. S., Gravenstein, N. & Gravenstein, D. *Essential Anesthesia: From Science to Practice* 2nd edn. (Cambridge University Press, 2011).

[31] Pifferi, A., Torricelli, A., Taroni, P. & Cubeddu, R. Reconstruction of absorber concentrations in a two-layer structure by use of multidistance time-resolved reflectance spectroscopy. *Opt. Lett.* **26**, 1963. 10.1364/OL.26.001963 (2001). 18059746 10.1364/OL.26.001963

[32] Beek, J. F., van Staveren, H. J., Posthumus, P., Sterenborg, H. J. C. M. & van Gemert, M. J. C. The optical properties of lung as a function of respiration. *Phys. Med. Biol.* **42**, 2263–2272. 10.1088/0031-9155/42/11/018 (1997). 9394411 10.1088/0031-9155/42/11/018

[33] Barthelemy, P., Bertolotti, J. & Wiersma, D. S. A Lévy flight for light. *Nature* **453**, 495–498. 10.1038/nature06948 (2008). 18497819 10.1038/nature06948

[34] Jacques, S. L. Corrigendum: Optical properties of biological tissues: A review. *Phys. Med. Biol.* **58**, 5007–5008. 10.1088/0031-9155/58/14/5007 (2013). 10.1088/0031-9155/58/14/5007 23666068

[35] Eggebrecht, A. T. *et al.* Mapping distributed brain function and networks with diffuse optical tomography. *Nat. Photonics* **8**, 448–454. 10.1038/nphoton.2014.107 (2014). 25083161 10.1038/nphoton.2014.107 PMC4114252

[36] Lai-Fook, S. J. Pleural mechanics and fluid exchange. *Physiol. Rev.* **84**, 385–410. 10.1152/physrev.00026.2003 (2004). 15044678 10.1152/physrev.00026.2003

[37] Di Sieno, L. *et al.* Breakthrough light harvesting in time-domain diffuse optics with 100 mm2 silicon photomultiplier. *Opt. Laser Technol.* **161**, 109228. 10.1016/j.optlastec.2023.109228 (2023). 10.1016/j.optlastec.2023.109228

[38] Di Sieno, L. *et al.* Time-domain diffuse optics with 8.6 mm2 fast-gated sipm for extreme light harvesting. *Opt. Lett.* **46**, 424–427. 10.1364/OL.413577 (2021). 33449045 10.1364/OL.413577

