# Supplementary material for: Initial non-invasive in vivo sensing of the lung using time domain diffuse optics
Source: Sci Rep. 2024 Mar 15;14:6343. doi: 10.1038/s41598-024-56862-0 (PMC11350160; doi:10.1038/s41598-024-56862-0)
Supplement: Supplementary file 1 — Supplementary Figures. [file 41598_2024_56862_MOESM1_ESM.pdf]

Initial non-invasive in vivo sensing of the lung using time domain diffuse optics

Antonio Pifferi[1,2], **Massimo Miniati**[3], **Andrea Farina**[2], **Sanathana Konugolu Venkata Sekar**[4], **Pranav Lanka**[4], **Alberto Dalla Mora**[1], **Giulia Maffeis**[1,*], **and Paola Taroni**[1,2]

[1]Politecnico di Milano, Dipartimento di Fisica, 20133, Milan, Italy
[2]IFN-CNR, Consiglio Nazionale delle Ricerche, Istituto di Fotonica e Nanotecnologie, 20133, Milan, Italy
[3]University of Florence, Department of Experimental and Clinical Medicine, 50134, Florence, Italy
[4]Biophotonics@Tyndall, IPIC, Tyndall National Institute, T12R5CP, Cork, Ireland
[*]giulia.maffeis@polimi.it

Supplementary information

The following figures are attached as supplementary figures to the main figures, showing the other protocol (Prot5) with 5s duration of each phase and 10 repetitions (the first one with normal breathing), and the non-refolded analysis.

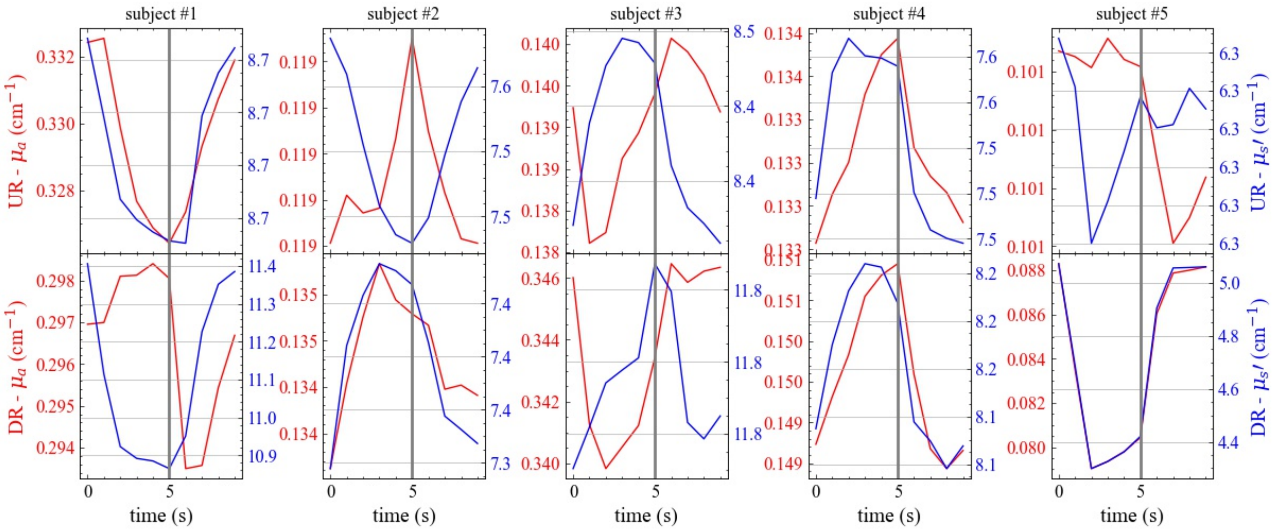

Supplementary Figure S1. In vivo time evolution of the absorption (red) and reduced scattering (blue) coefficients at 820 nm during the inhalation protocol Prot5 for the 5 volunteers (rows) and 2 locations (columns) over the lung. At $t = 0$ s, the subject is asked to inhale, while at $t = 5$ s the subject starts to exhale. Values refer to the folding average.

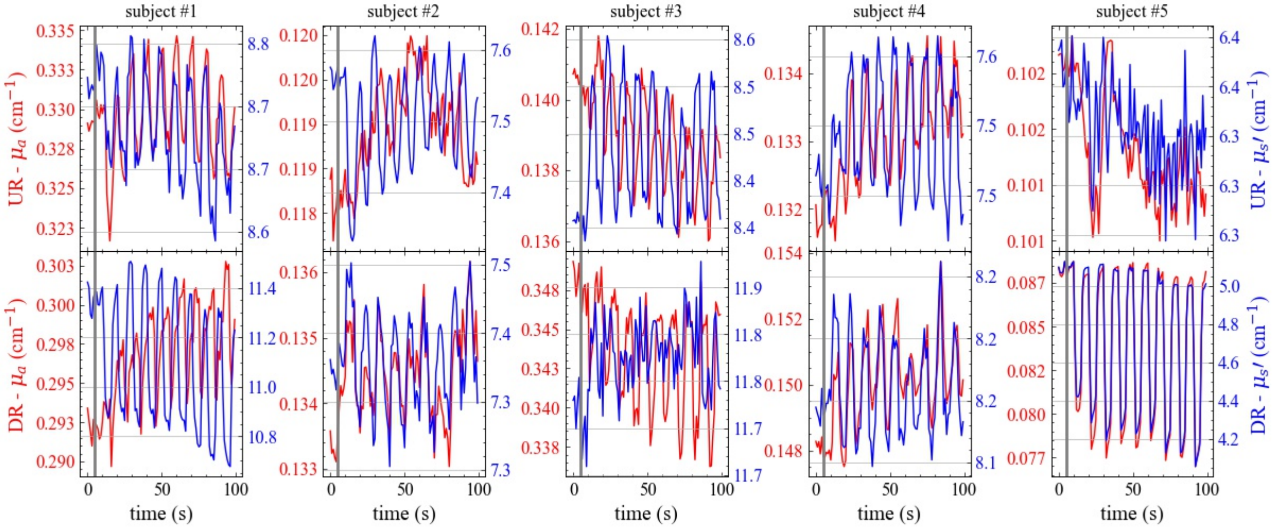

Supplementary Figure S2. Non-refolded in vivo time evolution of the absorption (red) and reduced scattering (blue) coefficients at 820 nm during the inhalation protocol Prot5 for the 5 volunteers (rows) and 2 locations (columns) over the lung.

.

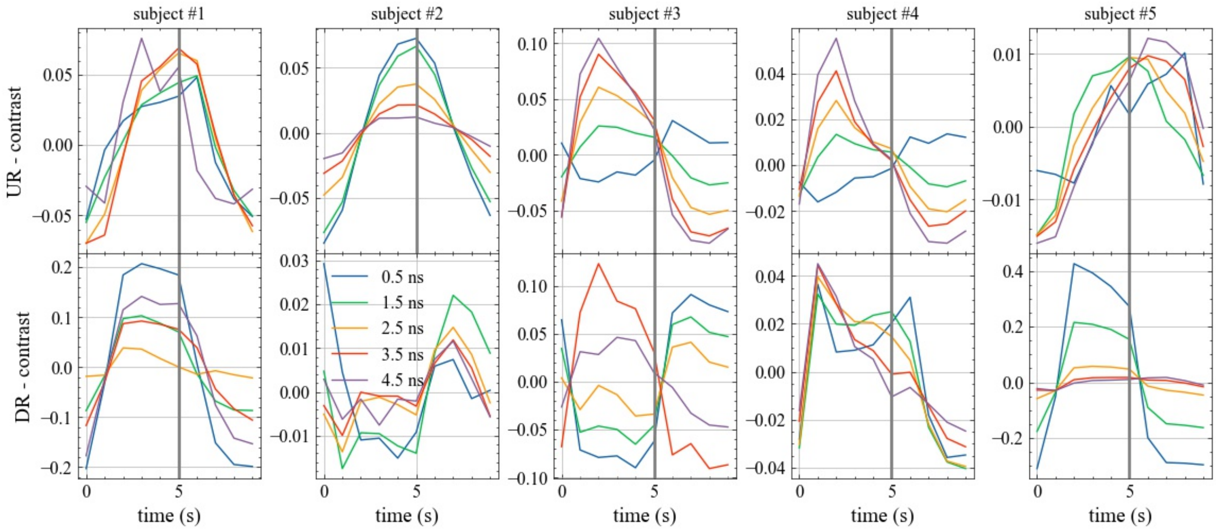

Supplementary Figure S3. In vivo time evolution of the relative contrast for different time gates (see legend) at 820 nm during the breathing protocol Prot5 for the 5 volunteers (columns) and 2 locations (rows) over the lung. At t = 0 s, the subject is asked to inhale, while at t = 5 s the subject starts to exhale. Values refer to the folding average.

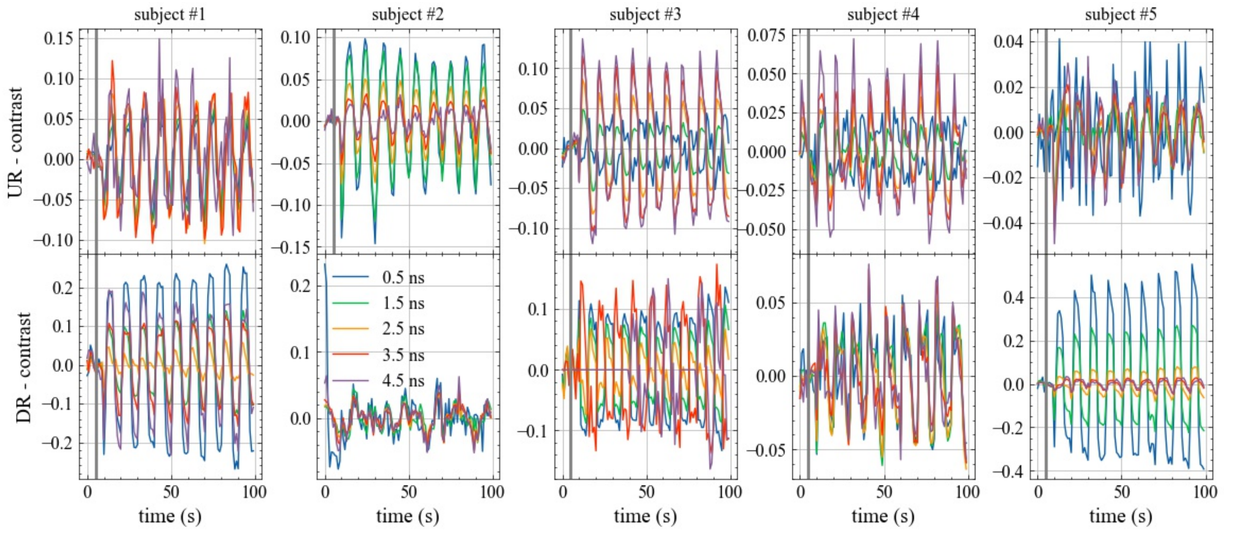

Supplementary Figure S4. Non-refolded in vivo time evolution of the relative contrast for different time gates (see legend) at 820 nm during the breathing protocol Prot5 for the 5 volunteers (columns) and 2 locations (rows) over the lung. At t = 0 s, the subject is asked to inhale, while at t = 5 s the subject starts to exhale.

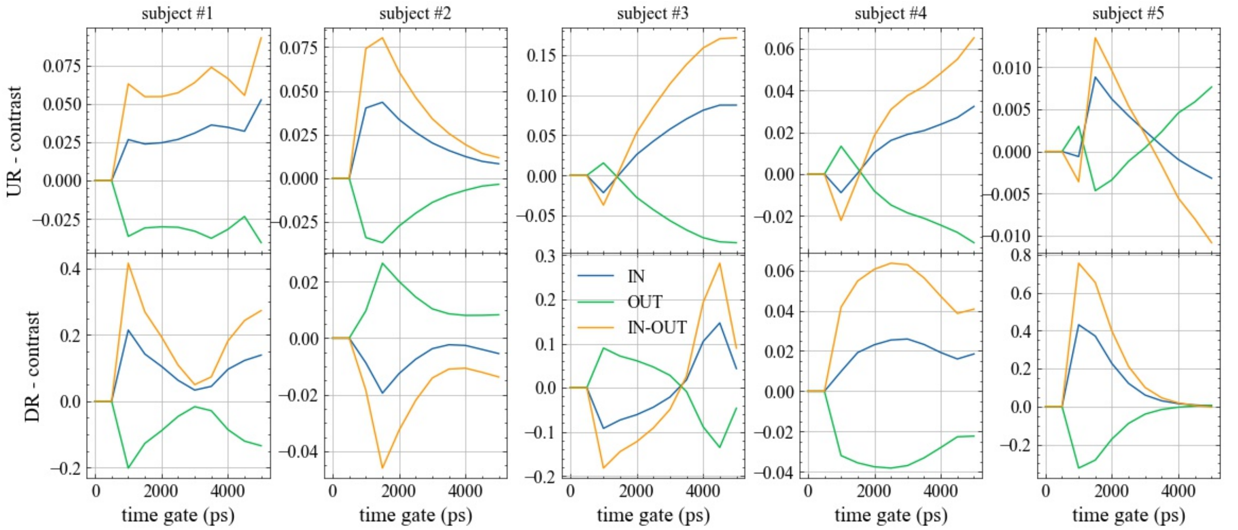

Supplementary Figure S5. Dependence of the relative contrast C vs photon propagation time during the plateau of the inhale (IN) or exhale (OUT) phases, together with the difference (IN-OUT) for the 5 volunteers (columns) and 2 locations (rows) for Prot5.
